# Effects of *Chenopodium album* L. Substitution Levels and Harvest Time on In Vitro Rumen Fermentation and Methane Production in Early-Fattening Hanwoo Steers

**DOI:** 10.3390/ani15101372

**Published:** 2025-05-09

**Authors:** Narantuya Batburged, Gui-Seck Bae, Gurbazar Damdinsuren, Sang-Yoon Kim, Hye-An Lee, Soo-Yeon Jung, In-Ki Kang, Da-Hyun Choi, Chang-Hyun Kim

**Affiliations:** 1 School of Animal Life Convergence Science, Hankyong National University, Anseong 17579, Republic of Korea; narantuyabat@naver.com (N.B.);; 2 Biogas Research Center, Hankyong National University, Anseong 17579, Republic of Korea; gsbae@hknu.ac.kr; 3 School of Animal Science and Biotechnology, Mongolian University of Life Sciences, Ulaanbaatar 17024, Mongolia; gurbazar@muls.edu.mn

**Keywords:** *Chenopodium album*, saponins, Hanwoo, rumen fermentation, methane production, substitution level

## Abstract

This study investigated the feasibility of incorporating *Chenopodium album* L. (CAL) into ruminant feed ingredients through evaluating the effects of harvest time and substitution levels on in vitro rumen fermentation. In the first phase, a sole-substrate experiment was conducted using CAL harvested from June to August, analyzing its chemical composition and total saponins content. The impact of harvest time on fermentation parameters was assessed with CAL as the sole substrate. The second phase involved a mixed-substrate experiment using an early-fattening Hanwoo diet (30% rice straw and 70% concentrate), where increasing proportions of CAL (control: 0%, T1: 5%, T2: 10%, T3: 15%, and T4: 20%) replaced rice straw. Seasonal variations in CAL composition influenced the fermentation characteristics. CAL harvested in July exhibited higher fermentability, with total volatile fatty acids (TVFAs) reaching 103.87 mM at 72 h. In contrast, CAL harvested in August showed lower fermentability and digestibility. However, August-harvested CAL was selected for the subsequent experiment, as it provided a more practical balance of sufficient biomass yield and a higher saponins concentration, aligned with the study’s methane mitigation objectives, while also exhibiting a fiber composition comparable to that of rice straw. We hypothesized that the saponins in CAL contribute to methane reductions. Supplementation with 15% of CAL significantly reduced methane production per gram of inoculated and digested dry matter (*p* < 0.05), likely due to differences in crude protein (CP), neutral detergent fiber (NDF), acid detergent fiber (ADF), and saponins content. However, despite having the lowest fiber content, T4 (20% CAL) exhibited the lowest in vitro dry matter digestibility (IVDMD), suggesting that factors such as saponins, CAL’s chemical composition, or microbial shifts may have hindered digestibility. Ammonia–nitrogen production increased from 0 to 3 h, but it continuously decreased between 3 and 9 h due to microbial growth and nitrogen assimilation, as microbes incorporate ammonia into their biomass (*p* < 0.05). Fermentation characteristics further revealed that the acetate-to-propionate (A/P) ratio decreased with increasing CAL levels, with T4 showing the lowest ratio (1.55 at 72 h), confirming a shift toward propionate-based fermentation. Notably, T2 (10% CAL) showed an optimized fermentation efficiency, producing the highest TVFA concentration at 24 h (98.28 mM). These findings highlight the potential for using CAL as a functional feed ingredient, with moderate substitution levels (10–15%) enhancing fermentation efficiency while reducing methane production.

## Simple Summary

This study investigated the use of *Chenopodium album* L. (CAL) in ruminant feed ingredients by evaluating the effects of harvest time and substitution levels on in vitro rumen fermentation. In the first phase, CAL samples harvested from June to August were analyzed for their chemical composition and saponins content, and their impact on fermentation was evaluated. In the second phase, CAL replaced rice straw in a Hanwoo diet at substitution levels of 0%, 5%, 10%, 15%, and 20%, and fermentation characteristics were measured. The results indicated that CAL harvested in July exhibited higher fermentability. However, CAL harvested in August was selected for the subsequent experiment, as it provided a more practical balance of sufficient biomass yield and a higher saponins concentration aligned with the study’s methane mitigation objectives, while also exhibiting a fiber composition comparable to that of rice straw. Supplementing the diet with 15% CAL reduced methane production, likely due to its chemical composition. The highest fermentation efficiency and optimal volatile fatty acid production occurred at a 10% substitution level. These findings suggest that the moderate substitution of CAL (10–15%) in ruminant diets could enhance fermentation efficiency and reduce methane production.

## 1. Introduction

Like any agricultural activity, cattle farming can have both positive and negative impacts on environmental sustainability. The environmental sustainability of cattle farming depends on several factors, including land use, water use, greenhouse gas (GHG) emissions, waste management, biodiversity conservation, antibiotic use, and social and economic impacts [1].

The emission of GHGs, such as carbon dioxide (CO_2_) and methane (CH_4_), ranks among the most important global environmental issues [2]. Ruminant livestock contributes approximately 15% to 20% of the total anthropogenic emissions of CH_4_ [3]. CH₄ produced from enteric fermentation in ruminants not only contributes to environmental problems but also represents a loss of energy, reducing its retention and utilization by the animal. Typically, 6% to 8% of the gross energy (GE) in animal feed is converted to CH_4_ during microbial digestion in the rumen, although up to 12% may be converted [4]. Reducing methane production from ruminants is therefore critical to lowering GHG emissions and improving the efficiency of the utilization of energy from digested feed.

In Korea, limited pasture areas, unfavorable soil and climate conditions, and high production costs create significant challenges for beef and dairy cattle farmers. The reliance on imported roughage, which is costly and often has a limited supply, places a financial burden on producers [5]. This situation is particularly challenging in countries like Korea and Japan, where beef cattle are raised for extended periods (e.g., up to 30 months). Consequently, there is increasing interest in reducing roughage feeding or finding cost-effective alternatives that maintain rumen health and productivity [5,6].

Considering these challenges, exploring alternative feed resources, such as *Chenopodium album* L. (CAL), has become increasingly important.

*Chenopodium album* L. (CAL), a native plant of Western Asia, belongs to the genus *Chenopodium*, which is distributed worldwide and contains about 250 species [7], with 15 species found in Republic of Korea [8]. CAL grows naturally in disturbed areas, salt marshes, and dry habitats [9].

While it has various ethnobotanical uses, including as a medicinal herb, its potential as a feed ingredient has received less attention. The green leaves of CAL are very nutritious and rich in essential amino acids, calcium, and vitamins A and C [10]. They are also a good source of fiber and iron content [11], with levels higher than those found in spinach and cabbage but lower than those observed in amaranth leaves [12].

CAL is rich in proteins (3.7% to 32.95%) and essential amino acids like lysine, leucine, and isoleucine [13], and contains bioactive compounds such as saponins [14], which have been shown to increase the digestibility of nutrients and reduce methane emissions in ruminants [15].

For example, saponins supplementation in diets for sheep increased the digestibility of organic matter (OM), neutral detergent fiber (NDF), and acid detergent fiber (ADF) by 9.6%, 27.9%, and 38%, respectively [16]. Some countries, such as Mongolia, use CAL as forage or silage [17] due to its high protein levels and bioactive contents [18]. CAL could be the cheapest and most readily available source of proteins, vitamins, minerals, fibers, and essential amino acids, especially for animal feed [19,20].

Therefore, this study aims to evaluate the feasibility of *Chenopodium album* L. (CAL) as a functional feed ingredient by examining the effects of harvest time and CAL substitution levels on in vitro rumen fermentation. The first objective is to assess how seasonal variations in the chemical composition and saponins concentration of CAL effect in vitro fermentation parameters when used as the sole substrate. The second objective is to investigate the impact of replacing rice straw with CAL at different levels (5%, 10%, 15%, and 20%) in an early-fattening Hanwoo diet (30% rice straw and 70% concentrate) on rumen fermentation characteristics, methane production, and nutrient digestibility. We hypothesize that CAL, particularly its nutrient composition and saponins content, can enhance fermentation efficiency and reduce methane production in ruminants.

## 2. Materials and Methods

### 2.1. Experimental Design, Feed Preparation, and In Vitro Fermentation Procedure

**Figure 1:**
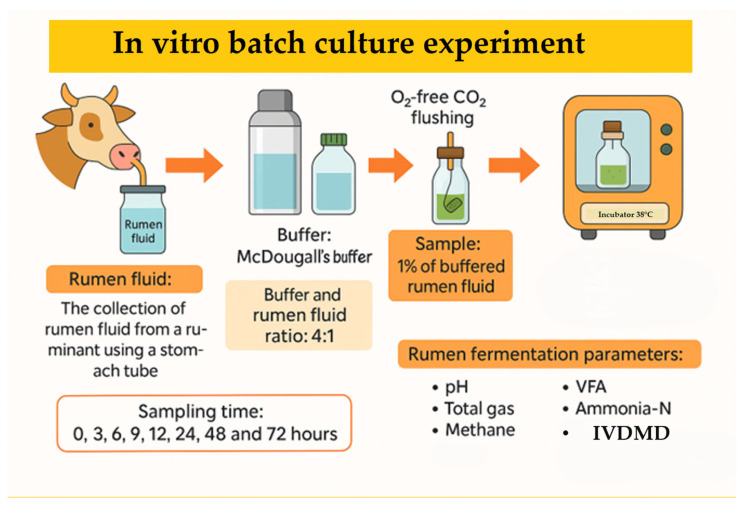
Procedure for the in vitro batch culture experiment.

We investigated the feasibility of incorporating *Chenopodium album* L. (CAL) into ruminant diets by evaluating the effects of harvest time and substitution levels on in vitro rumen fermentation. CAL was harvested at 30-day intervals between June to August from an agricultural field in Anseong, Republic of Korea (37°0′46.745″ N 127°17′28.218″ E). Test feed samples were prepared by drying fresh CAL at 60 °C in a drying oven (VISION, Daejeon, Republic of Korea) for 48 h, grinding the dried CAL with a hammer mill (micro-whisk mill, Culatti, Zurich, Switzerland) through a 1.5 mm sieve, and storing the powder in a –30 °C freezer (Gudero plus, Ilsinbio, Dongducheon, Republic of Korea) until further use. Seasonal variations in its chemical composition and total saponins content were analyzed. To evaluate its fermentation characteristics, we conducted a two-stage, in vitro batch culture experiment. In the first stage, we examined the impact of harvest time on fermentation parameters using CAL as a sole substrate. The second stage investigated the effects of replacing rice straw with increasing levels of CAL (0%, 5%, 10%, 15%, and 20%) in an early-fattening Hanwoo diet (30% rice straw and 70% concentrate; DM basis). August-harvested CAL, selected for its greater availability, was used as the rice straw substitute. The concentrate in the diet consisted of cornflakes (54.34%), corn gluten meal (5.08%), soybean meal (8.82%), wheat (6.51%), wheat bran (3.92%), palm kernel meal (6.55%), soybean hulls (6.51%), and lupin flakes (6.51%), along with limestone (0.77%), salt (0.37%), baking soda (0.37%), and Grobic-DC feed additive (0.25%). Rumen fluid was collected 30 min before the morning feeding from three Hanwoo steers using a custom-made stomach tube (Eco solution, Seoul, Republic of Korea) at the Chungnam National University research farm. The steers were fed a 50:50 (DM basis) diet of rice straw and concentrate (20.70% CP; 3.71% EE; 13.29% CA; 47.19% NDF; 39.71% ADF) (Approval No. 202404A-CNU-097). Approximately 1.5–1.6 L of rumen fluid steers was transferred into a 2 L thermos, filtered through four layers of cheesecloth, and diluted 1:4 (*v*/*v*) with McDougall’s buffer [21]. The diluted rumen fluid was dissolved with oxygen (O_2_)-free carbon dioxide (CO_2_) to make the fluid completely anaerobic; this was then used as an inoculum for the rumen. To a 120 mL serum bottle, 60 mL of buffered rumen fluid and 1% test feeds were added, sealed with a butyl rubber stopper and aluminum cap, and then cultured in a 39 °C incubator (HST-103M, Han Baek ST Co., Ltd., Yeoju, Republic of Korea) for 0, 3, 6, 9, 12, 24, 48, and 72 h (Figure 1). Following incubation, we analyzed the pH, in vitro dry matter digestibility (IVDMD), total gas production (TGP), methane production (CH_4_), ammonia–nitrogen production (NH_3_-N), and volatile fatty acids (VFAs).

### 2.2. Chemical Analysis and In Vitro Fermentation Measurements

Standard methods were used to analyze moisture, crude protein (CP), ether extract (EE), crude fiber (CF), and crude ash (CA) [22]. Non-fiber carbohydrates (NFC) were calculated using the summative equation method, which is commonly applied in ruminant nutrition studies [23]. Neutral detergent fiber (NDF) and acid detergent fiber (ADF) were analyzed according to Van Soest et al. [24]. The saponins content was analyzed by “Human bio” Food Analysis Research Center, using the method of Le Bot et al. [25]. The chemical compositions of experimental feeds and treatments used for in vitro batch culture experiment are presented in Table 1. We used a water displacement method, based on a method by Beuvink et al., to measure the TGP at each incubation time [26]. For CH_4_ analysis, we collected 4 mL of gas from the culture medium at specific incubation times. For the supernatant, we opened the serum bottle and extracted 10 mL of the culture media. We centrifuged the extracted media at 3000× *g* for 10 min using a centrifuge (Hanil Science, Gimpo, Republic of Korea) and collected the supernatant for further analysis. We measured the pH of the supernatant using a pH meter (Mettler, Columbus, OH, USA) and then stored the supernatant in a −30 °C deep freezer (IlshinBioBase, Dongducheon, Republic of Korea) for further analysis. Following centrifugation, we filtered the sediment and the remaining culture liquid into a glass crucible (FOSS, Hillerød, Denmark) and dried it at 105 °C for 4 h in a drying oven (VISION, Daejeon, Republic of Korea) before IVDMD was measured. A 2 mL aliquot of rumen fluid was mixed with 0.8 mL of 50% H_2_SO_4_ and 2 mL of ethyl ether to extract VFAs. The mixture was centrifuged at 3000 rpm (~1200× *g*) for 10 min. The clear supernatant (approximately 1.5–1.6 mL) was then passed through a 0.22 µm PTFE syringe filter before injection. One microliter of filtrate was injected onto a CP-Wax 58 FFAP CB capillary column (50 m × 0.53 mm; 2 µm film thickness) on a Shimadzu gas chromatograph (Kyoto, Japan) [27]. Gas chromatography, equipped with a thermal conductivity detector and a Hayesep Q packed column (Porapak-Q, 1.8 m × 2 mm i.d., 80/100 mesh size, Restek Corporation, Bellefonte, PA, USA), was used to determine CH_4_ concentration (Shimadzu, Kyoto, Japan). Prior to ammonia-N analysis, rumen samples were centrifuged at 10,000 rpm (≈10,000× *g*) for 5 min at 4 °C. The clear supernatant was then mixed with phenol color reagent (50 g phenol and 0.25 g sodium nitroferricyanide dihydrate, dissolved in 1 L distilled water) and alkali hypochlorite reagent (25 g NaOH and 16.8 mL sodium hypochlorite, diluted to 1 L). After incubating at 37 °C for 15 min to allow for color development, the reaction mixture was diluted with 8 mL distilled water, and absorbance was measured at 630 nm using a Thermo Scientific spectrophotometer (Waltham, MA, USA) [28].

### 2.3. Statistical Analysis

All data were analyzed using SAS® software (Version 9.4, SAS Institute Inc., Cary, NC, USA). The General Linear Model (GLM) procedure was employed to evaluate the effects of *Chenopodium album* L. (CAL) harvesting time and substitution level on in vitro rumen fermentation parameters at each incubation time. Tukey’s multiple comparison test was applied to determine significant differences between treatment means, with significance declared at *p* < 0.05. Superscripts (a, b, c, d, e) indicate statistically significant differences among treatments within the same incubation time. All values are presented as means along with their corresponding standard errors of the mean (SEM).

In addition, to evaluate the relationship between CAL substitution levels and the end-products of fermentation, *p*-values were calculated for each of the trend tests (linear, quadratic, cubic). A *p*-value of less than 0.05 was considered statistically significant. The assumptions of normality and homoscedasticity were not formally tested in this study [29].

## 3. Results

### 3.1. Chemical Composition and Saponins Content of Chenopodium album L. at Different Harvest Times

The chemical composition of CAL is presented in Table 2. The non-fiber carbohydrate (NFC) content was significantly higher (*p* < 0.05) in CAL harvested in July compared to that harvested in June and August. Although CAL harvested in July and August had similar levels of CP (12.49–13.24%) and EE (1.96–2.18%), the CP content of CAL harvested in June was statistically higher (*p* < 0.05). As the maturity of the CAL herb increased, the composition of CF, ADF, and NDF in CAL increased significantly (*p* < 0.05). In contrast, the CP content significantly decreased (*p* < 0.05).

The saponins content of CAL increased progressively as the plant matured, ranging from 6.60% in June to 7.05% in August (Table 3). Although this trend suggests a gradual accumulation of saponins over time, it is important to note that a statistical analysis cannot be conducted for these results. An external research center analyzed the saponin data without replication or statistical analysis.

### 3.2. Evaluation of the Effect of Harvest Time of Chenopodium album L. on In Vitro Rumen Fermentation

In Table 4, the pH levels of culture media with CAL harvested in July and August continuously decreased over 12 h of incubation. No significant differences were observed between the treatments at 3 and 9 h of incubation. At 6 and 12 h, the pH in July and August was lower than that in June (*p* < 0.05). After 24 h, the pH was lower in July compared to that in June and August (*p* < 0.05). At 48 h, the pH in July was the lowest among the treatments (*p* < 0.05), and at 72 h, the pH in June was significantly higher than that in July and August (*p* < 0.05). In Table 4, the IVDMD of culture media with CAL harvested in June was higher, while it was lower in August than that observed in July at all incubation times (*p* < 0.05). The IVDMD was negatively correlated with NDF and ADF content. In general, with advanced CAL plant maturity, there is an increase in the main features—DM, NDF, and ADF content—along with a decrease in CP content [30,31] and forage digestibility [32,33].

In Table 4, there are distinct temporal variations in NH_3_-N concentration across the three harvest treatments. The NH_3_-N levels increased from 0 h to 6 h, then declined between 9 h and 12 h, followed by an increase from 24 h onward. During the early fermentation phase (3 h, 6 h, and 9 h), no significant differences were observed among the harvest treatments. However, at 12 h, the culture media with CAL harvested in July reported the highest significant NH_3_-N concentration, while that harvested in June and August reported significantly lower values (*p* < 0.05). At 24 h, the NH_3_-N concentrations in June and August treatments were significantly higher than that in July (*p* < 0.05). After this point, NH_3_-N levels increased sharply, with the highest values observed in June, followed by July. The culture media with CAL harvested in August had the lowest concentrations at 48 h and 72 h (*p* < 0.05).

In Table 5, TGP was expressed per gram of dry matter incubated, while CH_4_ production was calculated per gram of dry matter incubated and per gram of digested substrate. The TGP of culture media with CAL harvested in August was significantly lower than that of other harvest treatments at 48 h and 72 h following incubation (*p* < 0.05). At 6 h and 9 h, there were no significant differences among harvest treatments, but from 12 h to 72 h, TGP in July was significantly higher than that in June and August (*p* < 0.05).

In Table 5, methane production differed significantly (*p* < 0.05) among harvest months and incubation times. At 3 h, the July samples showed the highest CH_4_ values, while June had the lowest. From 6 to 72 h, CH_4_ increased across all treatments, with July being consistently higher than June and August at most time points (*p* < 0.05). The samples from August showed intermediate values, but at later stages (48 and 72 h), these values were significantly lower than those obtained for July. When CH_4_ was expressed per gram of digested dry matter (DMD), clear differences were also observed (*p* < 0.05). August samples had the highest CH_4_ per g DMD mid-incubation, despite having lower overall digestibility. By 72 h, July maintained the highest CH_4_ per g DMD, followed by August, and then June.

In Table 6, the total VFA concentration was significantly higher in the June and July treatments compared to that in August at 0, 3, 6, 24, and 48 h of incubation (*p* < 0.05). At 9 h, the VFA concentration of culture media with CAL harvested in June was the lowest, while the VFA concentration was the highest in August (*p* < 0.05). Following 12 and 72 h of incubation, the highest total VFA concentration was observed in July (*p* < 0.05), indicating greater microbial fermentation activity. Acetate concentration was significantly higher in June at 3, 9, 24, and 72 h (*p* < 0.05), while no significant differences were observed among harvest treatments at 6 and 12 h. Propionate concentration did not differ significantly among the harvest groups from 0 to 6 h. However, for the harvest in July, propionate concentrations exhibited significantly higher values at 9, 12, and 24 h (*p* < 0.05). In the June and August harvests, propionate concentration was the highest, at 48 h (*p* < 0.05). The increase in total VFA production in June and July suggests enhanced microbial fermentation, likely due to the higher non-fiber carbohydrate content in younger CAL plants. The higher propionate levels in July suggest more efficient fiber fermentation, which aligns with the increase in total VFA production. Despite the higher NDF and ADF, the fermentation process may have favored propionate-producing pathways, such as the succinate-propionate pathway. Butyrate concentration was significantly higher in harvests in July and August at 0, 3, 12, and 72 h (*p* < 0.05). The highest butyrate concentration was observed at 9 h in the culture media with CAL harvested in July, while significantly higher values were obtained at 24 to 48 h in harvests in August (*p* < 0.05). The acetate to propionate ratio ranged from 3.02 to 3.66. Between the 3 to 6 and 12 to 24 h ranges, and at 72 h, a significantly lower acetate-to-propionate ratio was observed in July than that observed in the harvests in June and August (*p* < 0.05). This suggests a shift toward propionate fermentation. The effect of harvest time of sole-substrate *Chenopodium album* L. (CAL) on in vitro rumen fermentation parameters over time is shown in Figure S1 (Supplementary Materials). This includes (A) pH, (B) in vitro dry matter digestibility (IVDMD), (C) total gas production, (D) methane (CH₄) production, (E) total volatile fatty acids (TVFA), and (F) ammonia nitrogen (NH₃-N) concentration. All parameters demonstrated significant variation with harvest time. Values are presented as means ± SEM (n = 3).

### 3.3. Effect of Chenopodium album L. Substitution Levels on In Vitro Rumen Fermentation in Early-Fattening Hanwoo

Although CAL harvested in July exhibited higher fermentability, its lower biomass yield limited its suitability for practical application. In contrast, CAL harvested in August provided a more favorable balance, offering both an adequate biomass and a saponins concentration consistent with the study’s methane reduction objectives, while also displaying a fiber composition comparable to that of rice straw. Additionally, minimizing the differences in nutritional composition between the test and control forages was considered important, as substantial variation could confound the interpretation of fermentation outcomes. These characteristics made the August harvest a more appropriate candidate for the substitution trial. Notably, saponins content increased with plant maturity, reaching 7.047% in August, compared to 6.619% in July and 6.597% in June (Table 3).

In Table 7, the pH values varied significantly among different CAL substitution level groups and incubation times. The pH was higher in treatments at early incubation times compared to the control. Although the control showed the highest value, pH significantly declined by 12 h across all treatments, indicating greater acid production from microbial fermentation in the CAL-substituted diet groups. At 24 h, T4 had the lowest pH, while at 48 h, T1, T2, and T3 recorded the lowest pH values, suggesting a prolonged fermentation process, likely due to increased organic acid production. No significant differences were observed among treatments by 72 h, indicating that pH became stable with prolonged incubation. To further characterize the effect of CAL substitution levels on pH dynamics, a contrast analysis was performed. The results showed significant linear effects at most incubation times, quadratic effects from 3 to 24 h, and cubic effects at 6, 9, 12, and 48 h. No significant differences were observed among treatments at 72 h.

IVDMD varied significantly among treatments over time. At 3 h, T1 exhibited the highest digestibility (28.94%), while T4 showed the lowest (14.71%). This trend continued at 6 h, where T1 was not significantly different from T2 and T3, suggesting enhanced microbial activity with partial CAL substitution. Digestibility was highest in the control and T3 by 9 h, indicating the rapid degradation of more fermentable components. At 12 and 24 h, digestibility was significantly higher in T2 and T3 compared to that of other substitution treatments, suggesting improved fermentation efficiency. By 48 and 72 h, digestibility remained high across all treatments, with the highest values observed in T2 at 48 h. IVDMD was the lowest in T4 compared to other treatments at the same incubation time (*p* < 0.05). Linear effects were significant at all time points, while quadratic effects were significant at most times, except at 9 h. Significant cubic effects were also detected at 9, 12, 24, 48, and 72 h, indicating complex response patterns.

NH_3_-N concentration followed a dynamic pattern across incubation times. At 0 h, initial NH_3_-N levels were highest in T3, suggesting a high proportion of rapidly degradable proteins. By 3 h, T1 and T3 showed significantly increased NH_3_-N concentrations (*p* < 0.05), indicating rapid protein breakdown. At 6 h, the NH_3_-N concentration was the highest in T4, suggesting the continued deamination of dietary proteins. By 9 h, NH_3_-N was the lowest in T2 (*p* < 0.05), implying a slow release of ammonia or an increase in the assimilation of microbial nitrogen. At 12 h, NH_3_-N was the highest in the control but the lowest in T4 (*p* < 0.05). At 24 h, NH_3_-N was highest in T3, followed by T4, indicating prolonged protein degradation. By 48 and 72 h, NH_3_-N increased sharply across all treatments, with the highest values being observed in the control, T3, and T4, suggesting extensive protein breakdown and reduced microbial nitrogen utilization at later incubation times. In the in vitro experiments with either the CAL sole substrate or the diet supplemented with varied proportions of CAL, NH_3_-N concentrations initially increased from 0 to 3 h, decreased between 6 and 9 h, and then increased again after 12 h. These fluctuations can be attributed to key factors influencing rumen fermentation dynamics. In the early phase (0–3 h), rapid protein degradation and high microbial activity lead to increased NH_3_-N levels. Subsequently, NH_3_-N is reduced with microbial growth and nitrogen assimilation as microbes incorporate ammonia into their biomass. After 12 h, microbial turnover, the increased degradation of complex substrates, and the reduced efficiency of microbial protein synthesis contribute to the high NH_3_-N concentrations. Linear effects were significant at most times, except at 9 h, indicating a consistent trend over time, except at 9 h. Quadratic effects were significant at 0, 3, 12, 48, and 72 h, suggesting a curvilinear relationship at these times. Cubic effects were significant at 0, 3, 24, 48, and 72 h, indicating a more complex relationship with multiple changes in direction over these periods.

In Table 8, TGP was expressed per gram of incubated dry matter, while CH_4_ production was calculated per gram of both incubated dry matter and digested substrate. TGP and CH_4_ production varied significantly among different CAL substitution levels and incubation times (*p* < 0.05). At 3 h, T4 exhibited the highest TGP (*p* < 0.05), suggesting the rapid fermentation of readily available carbohydrates. However, at 6 h, T1 had the highest TGP, while by 12 h, TGP was significantly lower in T3 and T4 compared to the other treatments, indicating a reduced fermentation rate at higher CAL substitution levels. At 24 h, the control and T1 showed the highest TGP (*p* < 0.05), but by 72 h, all treatments exhibited similar values, suggesting fermentation stabilization over time. Linear effects were significant at most times, except at 9 h, indicating a generally consistent relationship between the variable and the outcome at most times, but not at 9 h. Quadratic effects were observed at 3, 12, and 24 h, showing a non-linear relationship where the effect changes direction at these times. Cubic effects were detected at 6, 9, 24, 48, and 72 h, indicating a more complex relationship with multiple changes in direction over time (*p* < 0.05).

Methane production per gram of DM varied significantly (*p* < 0.05) across treatments and incubation times. At 3 h, T2 and T4 showed higher CH_4_ values, while T3 consistently had the lowest CH_4_ production at 6, 12, and 72 h (*p* < 0.05). By 24 h, methane production increased across all treatments but remained significantly lower than the control. Contrast analysis revealed significant linear effects at 9 h and from 24 to 72 h (*p* < 0.05), a quadratic effect only at 24 h, and significant cubic effects at 6, 9, 12, and 72 h (*p* < 0.05).

Regarding CH_4_ production per gram of DMD, T1 and T3 exhibited the lowest CH_4_ output at 3 h (*p* < 0.05). T3 consistently showed the lowest CH_4_ production at 6, 9, 24, and 72 h (*p* < 0.05), while at 12 h, T2 had significantly lower CH_4_ production. CH_4_ production was highest in the control at 48 and 72 h, whereas the T4 showed the highest CH_4_ production at all incubation times. Linear effects were significant at most times, except at 24 and 48 h, suggesting a consistent trend over time except for at these specific time points. Quadratic effects were detected at most times, except at 72 h, with a curvilinear relationship being observed at these times. Cubic effects were observed from 3 to 24 and at 72 h, indicating further complexity in the relationship during these periods (*p* < 0.05).

In Table 9, at 0 h, all substitution levels had similar TVFA concentrations, except those observed in T3, in which levels were significantly lower than in the control (*p* < 0.05). By 3 h, TVFA production peaked in T4 and the control, while other treatments remained similar. At 9 h, T2 and T4 had the highest TVFA concentrations, indicating an increased fermentation rate. By 12 h, the control had the highest total TVFA (63.47 mM), but T3 and T4 showed comparable levels, suggesting no adverse effects occurred at higher proportions of CAL substitution. At 24 h, T2 had a significantly higher TVFA (98.28 mM) than the other treatments (*p* < 0.05), indicating that 10% CAL supplementation in the diet optimizes fermentation by providing an ideal nutrient balance. By 48 h, T4 had the highest TVFA (*p* < 0.05), while it was lower in the control, T1, and T3. At 72 h, the T4 (109.05 mM) had a significantly higher TVFA concentration than the control (102.91 mM), suggesting that high CAL levels support prolonged fermentation. Treatments showed varying patterns compared to the control, with linear effects not being significant at most times, except at 6 and 48 h. Quadratic effects were significant at 3 h, and from 12 to 48 h, and cubic effects were significant at 0, 6, 9, 12, and 48 h (*p* < 0.05). These trends indicate that CAL, particularly at 10% to 20% supplementation, provides fermentable substrates that sustain microbial activity.

At 0 h, acetate levels were comparable, although they were slightly lower in T4 (58.69%) than in the control (59.50%). By 3 h, T4 showed higher acetate values (60.04%) than the control (58.89%). At 9 h, acetate was significantly higher in T2, T3, and T4 (*p* < 0.05), with T1 and T2 peaking at 12 h (~59%), suggesting that a moderate level of supplementation (5–10%) promotes acetate production. Acetate percentages were significantly affected by treatment (*p* < 0.05) at several time points. Linear effects were significant at 0, 3, 9, 24 and 72 h, with quadratic effects observed at 0, 3, 9, 12 and 48 h. Cubic effects were detected at 6, 12 and 72 h (*p* < 0.05). Since acetate levels declined over time, and a higher level of CAL substitution (15–20%) shifted fermentation toward propionate, this suggests that a potential increase in fermentable carbohydrate proportion with increasing CAL supplementation could have contributed to this shift (Table 1). T1 and T4 had slightly higher propionate levels (16.30% and 16.19%) than the control (15.93%). The propionate increased significantly over time, peaking in T4 at 6 h (25.14%) and 9 h (25.13%). At 24 h, T3 and T4 had significantly higher propionate levels than the control (*p* < 0.05). By 48 and 72 h, T4 maintained the highest propionate concentration, supporting a sustained shift toward more efficient fermentation. Propionate is a major glucose precursor, which enhances energy efficiency and potentially reduces methane production as there is less hydrogen available for methanogenesis. The propionate percentages were significantly affected by treatments at most times (*p* < 0.05). Linear effects were significant at most times, except at 0, 9 and 12 h, and quadratic effects were detected at several time points, including at 3, 9, 12, and 48 h. The T4 treatment exhibited higher propionate percentages at 72 h compared to the control and other treatments. Cubic effects were significant, except at 3, 48 and 72 h, reflecting a complex response to the treatment levels.

The control had the highest A/P ratio (3.76), indicating an acetate-dominant fermentation. The A/P ratios for T1 (3.64) and T4 (3.69) were slightly lower. The ratio declined significantly over time, with T3 at 3.12 and T4 at 3.06 at 3 h. By 12 h, T1 had the highest A/P ratio (2.44), while the control was significantly lower (2.31). By 48 and 72 h, T4 had the lowest A/P ratio (1.55), confirming a shift toward propionate fermentation, which is often associated with reduced methane production and improved energy efficiency. The linear effects were significant at 3, 6, 9, 24, 48, and 72 h, while quadratic effects were observed at 3, 12, and 48 h. Cubic effects were significant at 0, 3, and 12 h (*p* < 0.05). Although IVDMD was reduced in T4, TGP was also lower in T4. Interestingly, CH_4_ mL/g DMD was the highest in T4, indicating that, despite the reduction in IVDMD, methane production per unit of digestible dry matter was greater in T4 compared to that in other treatments. The total VFA concentration remained unaffected, suggesting that the reduction in methane production was likely due to a shift in fermentation patterns rather than a change in overall fermentation activity.

Supplementation with 15% of CAL reduced methane production. The highest fermentation efficiency and optimal VFA production occurred at a 10% substitution level. However, despite having the lowest fiber content, T4 (20% CAL) exhibited the lowest IVDMD, suggesting that factors such as saponins, CAL’s chemical composition, or microbial shifts may have hindered digestibility. The effect of *Chenopodium album* L. substitution levels on in vitro rumen fermentation parameters over time is shown in Figure S2 (Supplementary Materials). This includes (A) pH, (B) in vitro dry matter digestibility (IVDMD), (C) total gas production, (D) methane (CH₄) production, (E) total volatile fatty acids (TVFA), and (F) ammonia nitrogen (NH₃-N) concentration. All parameters demonstrated significant variation with substitution levels. Values are presented as means ± SEM (n = 3).

## 4. Discussion

### 4.1. Chemical Compositions and Saponins Content of Chenopodium album L. at Different Harvest Times

As the maturity of the CAL herb increased, the composition of CF, ADF, and NDF in CAL increased significantly (*p* < 0.05). In contrast, the CP content significantly decreased (*p* < 0.05). The chemical composition of forage has a critical influence on its nutritive value, with protein and fiber concentrations serving as key indicators of feed quality [34]. Adedapo et al. [35] and Han et al. [8] reported CP levels of 26.44% and 28.72%, respectively, in CAL leaves, which are higher than the values obtained in our study. Singh et al. [36] and Odhav et al. [37] reported CP contents of 3.7% and 5.0%, respectively, in CAL, which are lower than the values observed in our current study. Peiretti et al. [38] observed that the CP content of *Chenopodium quinoa* ranged from 9.35% at the budding stage to 15.1% at the grain stage, while the NDF content ranged from 40.8% at the mid-vegetative stage to 53.4% at the grain stage. These data align with the results of our study. Afolayan et al. [39] reported that the CP content of the vegetables they examined ranged from 13.25% to 26.44%, with the highest CP content observed in CAL and the lowest in *Sonchus asper*. The protein content in these wild species was observed to be comparable to or higher than that of exotic vegetables, such as lettuce, cabbage, and spinach [39]. Similar findings have been reported in Nigeria and the Coruh Valley where the protein content of wild vegetables ranged between 3.5% and 32.5% [40,41]. With its high crude protein content, CAL could serve as an affordable protein source for livestock.

Saponins synthesis in plants is influenced by both intrinsic and external factors, including physiological stage, cutting time, and environmental conditions, such as soil type and climate [42]. The progressive increase in saponins content observed in our study may be partially attributed to the rise in average temperatures during the experimental period; temperatures were 28.1 °C in June, 30.2 °C in July, and 31.3 °C in August [43]. Saponins content was generally lower in early spring and mid-autumn [44]. The growth stage of plants also plays a crucial role, with saponins levels typically peaking at the blooming stage and declining during the grain stage. For instance, alfalfa’s saponins content ranges from 0.1% to 2.5% [45] depending on the variety and is the highest when harvested in late summer [44]. Similarly, *Chenopodium quinoa*, a closely related species, often exhibits high saponins levels, with levels in certain varieties exceeding 10% [46]. In contrast, commonly used forage crops, such as alfalfa and soybeans, have significantly lower saponins content, averaging 4.0% and 1.4%, respectively [47]. The relatively high saponins content in CAL highlights its potential as a forage crop with bioactive properties. However, caution is warranted, as excessive saponins intake can lead to antinutritional effects, including reduced feed palatability and potential toxicity in livestock. The lethal dose of saponins varies widely, ranging from 25 to 3000 mg/kg body weight, depending on the animal species and the chemical structure of the saponins [47]. Future studies should evaluate the optimal supplementation levels of CAL in animal feeds to maximize its benefits while minimizing the potential risks. Although no correlation analysis was conducted in the present study, previous research has demonstrated significant relationships between plant chemical composition and fermentation parameters [48], supporting the possibility of such interactions in the current findings.

### 4.2. Evaluation of the Effect of Harvest Time of Chenopodium album L. on In Vitro Rumen Fermentation

The lower pH observed in July and August, despite the higher NFC content in July, may reflect a reduced buffering capacity in more mature plants. Younger plants harvested in June likely possessed greater buffering potential due to their higher concentrations of organic acids, minerals, and soluble nitrogen compounds, which can moderate the early pH decline during fermentation. As plants mature, soluble carbohydrate availability declines while structural fiber content increases, resulting in delayed but more intense acid production as fermentation progresses [24,49].

The IVDMD was negatively correlated with NDF, ADF content, and maturity, indicating that as fiber content and plant maturity increased, the digestibility of the dry matter decreased. Balde et al. [50] performed in vivo experiments using four mature stages of fresh alfalfa and found that the effective degradation of dry matter reduced with increasing plant maturity. Yu et al. [51] reported that alfalfa hay harvested at the early budding and late budding stages had higher IVDMD and NDF digestibility than when the alfalfa hay was harvested at the early flowering stage [51]. The IVDMD in CAL was compared to that of common forages, such as rice straw and timothy hay. Rice straw has low digestibility (37.2%) [52] due to its high fiber content and silica [53], making it less suitable for high-yield animals. Timothy hay has a moderate IVDMD (52.2%) depending on its fiber composition [54]. The IVDMD of culture media with CAL harvested in June and July (72.14–82.62%) was higher than that of both rice straw and timothy hay, indicating superior fermentability and nutrient availability. A slight decrease in IVDMD at 72 h compared to 48 h in July- and August-harvested CAL forages may be attributed to microbial colonization and biomass accumulation on undigested residues. During extended incubations, fibrolytic microbes form biofilms and contribute microbial mass to the residue, potentially leading to an underestimation of digestibility when using IVDMD. This limitation could have been addressed by measuring IVTDMD [55].

Similar trends in NH_3_-N accumulation over time have been reported in studies on the ruminal degradation of protein-rich forages [56]. NH_3_-N serves as an indicator of protein degradation in the rumen, where the microbial deamination of dietary proteins releases ammonia, which can be utilized for microbial protein synthesis or absorbed into the bloodstream [57]. The increased fiber content and low soluble protein fractions that are observed as the plant matures may suggest reduced protein degradation, which lead to lower NH_3_-N concentrations in the rumen mix, with CAL harvested in August [56]. Higher NH_3_-N levels in June and July indicate the rapid breakdown of protein, possibly due to the greater CP availability in young plants. This observation is consistent with previous findings on forage maturity and nitrogen release [58].

TGP is an indicator of the efficiency of microbial fermentation. The higher TGP in the CAL harvest in July suggests the greater availability of fermentable carbohydrates compared to CAL harvests in June and August. Fermentable carbohydrates may have resulted in enhanced microbial activity, leading to an increase in TVFA production in July. Furthermore, a positive relationship between total gas volume and TVFA production was observed 12 to 72 h following incubation. This finding aligns with the results reported by Muck et al. [59] and Blümmel et al. [60], who also noted that high gas production rates correspond to increased fermentation and VFA production. Significant differences (*p* < 0.05) in CH_4_ production highlight the impact of harvest time on fermentation. The July samples produced the most CH_4_, likely due to the increase in fermentable substrate content. In contrast, August showed an elevated CH_4_ per g DMD despite the lower overall CH_4_, possibly due to reduced digestibility inflating the CH_4_ yield per unit of digested matter. The June samples consistently showed the lowest CH_4_, suggesting better potential for methane mitigation. These results align with those of Johnson et al. [61], who linked increased fiber fermentation with higher methane output, and support the earlier findings [62] that plant maturity may influence methane dynamics over time.

Over time, TVFA production peaked between 48 and 72 h, with the most efficient fermentation being seen in July, followed by June. The slowest fermentation rate was observed in August; however, the higher total TVFA concentration at 9 h suggests that certain fiber-degrading microbes were more active in the August treatment, leading to an increase in TVFA production at this specific time. These results are generally consistent with previous findings on the forage fermentation dynamics in cattle [63].

Topps et al. [63] previously reported that butyrate proportions remain relatively stable with advanced forage maturity, which is consistent with the trends observed in this study. Lower acetate-to-propionate ratios are associated with increased energy-efficiency and are favorable for high rates of beef production [58]. Increased propionate fermentation is considered more energy-efficient than elevated acetate fermentation [64], as increased propionate production enhances the utilization of acetate at the tissue level and improves nitrogen retention [65]. In the present experiment, TGP, IVDMD, and total VFA production were low at later stages of plant maturity (August). This decline in fermentation efficiency was likely due to a decrease in nutritional value, and, particularly, an increase in the fiber content, as previously reported in similar studies [66].

In the experiment using CAL as the sole substrate, there was minimal variation in saponins concentrations across treatments in each serum bottle. Significant differences in CP and fiber content were observed, which may be related to the lower digestibility of CAL harvested in August. Despite the higher saponins content observed in the August harvest, no significant effect of saponins on methane production was observed. In fact, methane production was higher in August compared to June and July, suggesting that factors other than saponins content, such as CP and fiber composition, may influence fermentation and methane production. It is important to note that this study was based on CAL collected from a single location in a single harvest year. Environmental factors such as climate and soil conditions may affect chemical composition, saponins content, biomass yield, and fermentability. Therefore, future studies should validate these results across multiple locations and growing seasons and consider using a CAL with higher fermentability to optimize rumen fermentation efficiency.

### 4.3. Effect of Chenopodium album L. Substitution Levels on In Vitro Rumen Fermentation in Early-Fattening Hanwoo

Saponins are known to influence rumen fermentation by reducing methane production and altering microbial populations, potentially improving protein utilization [67]. However, excessive saponins intake may have antinutritional effects, such as reducing feed intake or impairing microbial activity [68]. The moderate increase in saponins content in August could offer some functional benefits in rumen fermentation. Although the August harvest of CAL may not provide the same optimal nutritional profile as the July harvest, its inclusion provides a practical assessment of CAL’s potential as a viable feed substitute through considering variations in chemical composition across different harvest periods.

The IVDMD results showed that moderate CAL levels (T1, T2 and T3) improved digestibility, but high substitution levels (T4) consistently had lower digestibility, suggesting that at higher inclusion levels (20%), factors such as secondary metabolites like saponins and tannins could limit microbial access to nutrients [48].

NDF and ADF decreased with increasing CAL substitution rates, which suggests improved digestibility. The lower fiber content observed in CAL suggests an improvement in digestibility parameters compared to traditional forages like rice straw [69,70]. The observed decrease in NDF and ADF with higher CAL substitution rates illustrates the enhanced nutrient availability for the microbes within the rumen environment, subsequently improving fermentation efficiency [71]. This complementarity of feed substitutes like CAL illustrates their function as viable alternatives to traditional feeds. The results suggest that low to moderate substitution levels of CAL (5–15%) can enhance or maintain IVDMD compared to the control, while high substitution levels (20%) negatively impact digestibility. The significant linear and quadratic responses indicate a predictable pattern where moderate inclusion benefits fermentation, but excessive levels hinder it. The significant cubic effects reflect the more complex fermentation dynamics, especially at later stages of incubation. The higher IVDMD observed in sole-substrate incubations compared to mixed diets containing 70% concentrate during early incubation may be attributed to differences in the substrate composition. In the mixed diets, the presence of highly fermentable starch could have influenced microbial substrate preference or temporarily delayed fiber degradation due to competitive nutrient utilization. Although microbial populations and fiber-specific digestion were not directly measured in this study, the observed pattern aligns with associative effects described in previous studies involving mixed forage–concentrate systems [72,73].

The pH dynamics during fermentation signify meaningful alterations in microbial activity and fermentation patterns. A significant drop in pH across treatments indicates robust organic acid production, which is primarily attributed to the activity of fermentative bacteria [74].

Regarding NH_3_-N levels, early fermentation phases exhibited higher concentrations compared to controls, suggesting efficient protein breakdown [75]. As fermentation progressed, NH_3_-N levels declined, likely due to the enhanced microbial assimilation of nitrogen [76]. This trend underscores the importance of moderation when incorporating saponin-rich feeds to optimize rumen function and avoid excessive protein degradation that could lead to ammonia accumulation—an undesirable outcome in ruminant nutrition [77]. Studies have documented that ammonia concentrations beneath this minimum threshold indicate potential for uptake by rumen microbes. These findings indicate that the moderate inclusion of CAL may enhance rumen nitrogen efficiency during fermentation. Studies have indicated that NH_3_-N concentrations below a certain threshold may reflect efficient microbial uptake. Miguel et al. [78] reported that low NH_3_-N levels can result from the rapid utilization of newly released nitrogen for microbial protein synthesis. This suggests microbial adaptation to efficiently utilize available ammonia, potentially improving fermentation processes. Such dynamics play a crucial role in nutrient utilization by ruminants, as supported by studies examining microbial protein synthesis under varying nitrogen conditions [79,80].

Nevertheless, while low NH_3_-N concentrations demonstrate that microbes assimilate available nitrogen efficiently, the absence of directly measured microbial biomass or protein synthesis limits the interpretation of these results.

Total gas production (TGP) was highest in T4 at 3 h, suggesting rapid fermentation at the early stage. However, by 12 h, TGP was lower in T3 and T4, which may be attributed to the effects of saponins on rumen microbial dynamics. Saponins are known to suppress protozoa and methanogenic archaea [68,81], leading to reduced methane and total gas production. They may also inhibit fiber-degrading bacteria, potentially lowering IVDMD without significantly affecting TVFA output [82]. Furthermore, saponins can shift fermentation toward propionate production, a more energy-efficient pathway associated with a reduced gas output [83]. Therefore, the reduced gas production in T4 likely reflects altered microbial activity and fermentation patterns rather than diminished overall fermentative efficiency [71].

T3 (15% CAL) consistently had the lowest levels of CH_4_ production, supporting a shift toward more efficient propionate fermentation. Interestingly, T4 showed an increase in CH_4_ production per gram of digested dry matter (DMD), despite the lower in vitro dry matter digestibility (IVDMD), likely due to a mathematical artifact wherein reduced digestibility lowers the denominator, artificially inflating CH_4_ production per gram of DMD. When expressed per gram of incubated dry matter (DM), T4 exhibited significantly higher CH_4_ production at 3 and 6 h (*p* < 0.05), suggesting a more rapid early-phase fermentation. However, from 24 to 72 h, CH_4_ levels in T4 were comparable to those in other treatments and remained lower than the control, indicating that total methane production was not actually elevated. Additionally, propionate and TVFA concentrations were not reduced in T4, suggesting that fermentation efficiency was maintained.

The observed lower IVDMD in T4 may be attributed to the inhibitory effects of saponins on rumen protozoa and methanogenic archaea, which play a key role in fiber degradation. Their suppression likely impaired fiber digestion without significantly affecting bacterial fermentation or VFA synthesis. Notably, in T2 (10% CAL), the highest TVFA concentration at 24 h indicated optimal fermentation. In contrast, higher CAL substitution (T4) promoted a shift toward propionate production, reducing the acetate-to-propionate (A/P) ratio over time. This shift may explain the decreased acetate concentration in T4, which is linked to the observed reduction in IVDMD.

Saponins have been widely studied for their role in mitigating methane production and altering microbial populations in the rumen. They are believed to reduce methane emissions by interacting with rumen protozoa and methanogenic bacteria, leading to a more favorable fermentation profile that emphasizes propionate production over methane [84,85]. The inclusion of saponins could, therefore, enhance protein utilization by redirecting available energy from methane production toward microbial growth, which may have important implications for ruminant nutrition [86]. Evidence suggests that incorporating moderate levels of *Chenopodium album* (10–15%) optimizes digestibility, fermentation efficiency, and microbial nitrogen utilization, while simultaneously minimizing methane emissions [87]. However, inclusion levels of 20% or higher may disrupt rumen fermentation by decreasing IVDMD and fiber degradability, likely due to the inhibitory effects of excessive saponins on protozoa and methanogenic archaea. The effects of saponins appear to be dose-dependent, with excessive intake associated with inhibited microbial activity, leading to potential negative outcomes such as reduced feed intake and impaired fermentation dynamics [84].

While this study focused on saponins content, it is important to note that *Chenopodium album* contains other secondary metabolites, such as tannins, flavonoids, and alkaloids, which may also influence fermentation dynamics and gas production [88]. For example, dietary tannins have been reported to affect ruminal fermentation, improve protein utilization, and enhance animal performance parameters [89]. Tannins have also been associated with improved quality in animal products, such as milk and meat [48]. Although microbial and enzymatic analyses were not conducted in this study, it is possible that the combined effects of multiple phytochemicals in *Chenopodium album* contributed to the observed fermentation responses. Future research incorporating a broader phytochemical analysis and microbial profiling would help to clarify the mechanisms involved. From a practical standpoint, incorporating 10–15% *Chenopodium album* into ruminant diets appears promising as a sustainable methane mitigation strategy without compromising fermentation efficiency. However, further in vivo studies are necessary to ensure its safety, palatability, and long-term effectiveness under real feeding conditions.

## 5. Conclusions

The findings of this study provide compelling evidence that both harvest time and the proportional substitution of Chenopodium album L. (CAL) in ruminant diets significantly affect in vitro rumen fermentation processes, methane production, and overall nutrient digestibility. Specifically, the August-harvested CAL, which exhibited higher saponins levels and increased structural carbohydrate content, was associated with reduced digestibility and a decrease in overall fermentation efficiency. Despite this suboptimal nutritional profile, the greater availability of the August-harvested CAL justified its selection for dietary substitution, highlighting its practical applications in ruminant feeding strategies.

The assessment of CAL substitution levels revealed that increasing CAL levels in the diet was associated with a significant reduction in methane production (*p* < 0.05), with the 15% CAL substitution achieving the most pronounced reduction. Low-level CAL substitutions (5% and 10% CAL, respectively) optimized fermentation efficiency, leading to the highest total volatile fatty acid (VFA) concentrations at the 24 h mark. Notably, the decreasing acetate-to-propionate ratio with higher levels of CAL indicates a metabolic shift toward more energetically favorable propionate production, thereby enhancing the conversion efficiency of dietary energy for microbial growth.

Although CAL substitutions of up to 20% were included in this study, the appropriate level of CAL to incorporate into animal feed may depend on its chemical composition, particularly the concentration of secondary metabolites such as saponins and tannins. These compounds can influence the effectiveness of CAL in ruminant diets, especially regarding methane mitigation and fermentation efficiency.

In conclusion, substituting up to 15% CAL in ruminant diets can significantly improve rumen fermentation efficiency while mitigating methane emissions. However, higher substitutions (up to 20% CAL) may still provide benefits regarding prolonged fermentation dynamics, although at the cost of lower digestibility. These findings underscore the need for further research to investigate the long-term impacts of CAL dietary inclusion on animal performance, nutrient absorption, and overall health in vivo. Comprehensive evaluations involving various animal production systems and nutritional strategies will be crucial to optimize the use of CAL as a functional feed component in ruminant nutrition.

## Figures and Tables

**Table 1:** Chemical composition of individual feed ingredients and treatment diets for the second in vitro batch culture experiment.

Treatment/Feed	Ingredients (%)			Chemical Composition (% of DM)					
	Rice Straw	CAL	Concentrate	NFC ^1^	Crude Protein	Ether Extract	Crude Ash	NDF ^2^	ADF ^3^
Control (C)	30	0	70	38.47	13.96	6.20	6.65	41.80	21.92
T1	25	5	70	38.90	14.40	6.21	6.50	40.83	21.25
T2	20	10	70	39.33	14.83	6.23	6.34	39.87	20.59
T3	15	15	70	39.77	15.26	6.25	6.19	38.90	19.92
T4	10	20	70	40.20	15.69	6.27	6.03	37.93	19.25
Rice straw				18.58	3.87	1.82	7.50	73.34	47.22
CAL (August)				27.24	12.49	2.18	4.57	54.01	33.87
Concentrate				46.99	18.29	8.07	6.29	28.28	11.08

^1^ Non-fiber carbohydrate; ^2^ neutral detergent acid; ^3^ acid detergent acid.

**Table 2:** Chemical composition of *Chenopodium album* L.

Harvested Time	Moisture (%)	NFC ^1^	Crude Protein	Ether Extract	Crude Ash	Crude Fiber	NDF ^2^	ADF ^3^
		(% of DM)						
June	80.83 ^a^	30.44 ^b^	18.73 ^a^	4.92 ^a^	4.57 ^a^	17.36 ^c^	43.04 ^c^	21.69 ^c^
July	79.52 ^b^	38.45 ^a^	13.24 ^b^	1.96 ^b^	3.71 ^b^	18.78 ^b^	43.95 ^b^	27.53 ^b^
August	62.50 ^c^	27.24 ^c^	12.49 ^b^	2.18 ^b^	4.40 ^a^	26.09 ^a^	54.01 ^a^	33.87 ^a^
SEM ^4^	0.36	0.49	0.36	0.13	0.13	0.24	0.22	0.21
*p*-Value	<0.0001	<0.0001	<0.0001	<0.0001	<0.01	<0.0001	<0.0001	<0.0001

^1^ Non fiber carbohydrate; ^2^ neutral detergent acid; ^3^ acid detergent acid; ^4^ standard error of the means; superscripts (a, b, c) indicate statistically significant differences among treatments within the same incubation time.

**Table 3:** Saponins content of *Chenopodium album* L. and second experimental diets.

Harvest Time	Saponins (%)
June	6.60
July	6.62
August	7.05
Treatment ^1^	
Control	0.00
T1	0.21
T2	0.42
T3	0.63
T4	0.85

^1^ Substitution levels of *Chenopodium album* harvested in August by percentage: control (0), T1 (5), T2 (10), T3 (15), and T4 (20).

**Table 4:** Effect of *Chenopodium album* L. sole substrates on pH, in vitro dry matter digestibility (IVDMD), and ammonia–nitrogen (NH_3_-N) concentration.

Incubation Time (h)	Treatments ^1^			SEM ^2^	*p*-Value
	June	July	August		
pH					
0	7.01 ^a^	7.01 ^a^	6.99 ^b^	0.005	0.037
3	6.77	6.77	6.79	0.008	0.185
6	6.83 ^a^	6.73 ^b^	6.71 ^b^	0.014	0.002
9	6.69	6.68	6.70	0.006	0.010
12	6.72 ^a^	6.67 ^b^	6.65 ^b^	0.011	0.010
24	6.65 ^b^	6.63 ^b^	6.67 ^a^	0.004	0.003
48	6.70 ^b^	6.65 ^c^	6.75 ^a^	0.006	<0.0001
72	6.78 ^a^	6.71 ^b^	6.74 ^b^	0.011	0.008
IVDMD (%)					
3	38.09 ^a^	37.31 ^b^	31.92 ^c^	0.055	<0.0001
6	41.06 ^a^	38.82 ^b^	30.87 ^c^	0.160	<0.0001
9	46.50 ^a^	41.76 ^b^	35.76 ^c^	0.181	<0.0001
12	55.32 ^a^	45.93 ^b^	40.49 ^c^	0.916	<0.0001
24	72.87 ^a^	68.48 ^b^	52.35 ^c^	0.157	<0.0001
48	77.64 ^a^	73.59 ^b^	62.00 ^c^	0.186	<0.0001
72	82.62 ^a^	72.14 ^b^	60.99 ^c^	0.209	<0.0001
NH_3_-N (mg/100 mL)					
0	2.47 ^a^	1.57 ^c^	1.83 ^b^	0.058	<0.0001
3	3.8	3.7	3.53	0.090	0.189
6	3.4	3.47	3.27	0.079	0.269
9	2.8	2.6	2.9	0.094	0.152
12	2.40 ^b^	2.60 ^a^	2.23 ^b^	0.050	0.007
24	4.70 ^a^	3.56 ^b^	4.96 ^a^	0.250	0.015
48	11.00 ^a^	11.00 ^a^	8.97 ^b^	0.366	0.012
72	17.50 ^a^	15.30 ^b^	14.10 ^c^	0.111	<0.0001

^1^ Sole substrates of *Chenopodium album* according to harvested time: June, July, and August; ^2^ standard error of the means; superscripts (a, b, c) indicate statistically significant differences among treatments within the same incubation time.

**Table 5:** Effect of *Chenopodium album* L. sole substrates on in vitro total gas (TGP) and methane (CH_4_) production.

Incubation Time (h)	Treatments ^1^			SEM ^2^	*p*-Value
	June	July	August		
TGP (mL/g DM ^3^)					
3	10.59 ^b^	12.22 ^a^	10.22 ^b^	0.321	0.010
6	18.96	19.96	19.46	0.415	0.303
9	24.13	22.18	21.34	0.925	0.171
12	40.06 ^b^	43.89 ^a^	42.11 ^ab^	0.643	0.016
24	79.91 ^b^	91.68 ^a^	75.78 ^b^	1.490	0.001
48	104.44 ^b^	122.60 ^a^	81.24 ^c^	1.668	<0.0001
72	119.74 ^b^	136.52 ^a^	107.17 ^c^	1.518	<0.0001
CH_4_, (mL/g DM ^3^)					
3	0.22 ^c^	0.38 ^a^	0.27 ^b^	0.009	<0.0001
6	0.57 ^c^	0.79 ^b^	0.93 ^a^	0.029	0.0003
9	1.16 ^b^	1.23 ^b^	1.58 ^a^	0.045	0.0011
12	2.89 ^c^	3.57 ^a^	3.26 ^b^	0.050	0.0002
24	8.86 ^c^	10.68 ^a^	9.46 ^b^	0.053	<0.0001
48	13.06 ^b^	18.34 ^a^	11.27 ^c^	0.250	<0.0001
72	20.07 ^b^	21.03 ^a^	16.25 ^c^	0.165	<0.0001
CH_4_, (mL/g DMD ^4^)					
3	0.61 ^c^	1.00 ^a^	0.88 ^b^	0.009	<0.0001
6	1.35 ^c^	2.03 ^b^	2.93 ^a^	0.076	<0.0001
9	2.33 ^c^	3.01 ^b^	3.97 ^a^	0.074	<0.0001
12	5.46 ^c^	6.54 ^b^	8.04 ^a^	0.154	<0.0001
24	12.33 ^c^	15.88 ^b^	18.31 ^a^	0.285	<0.0001
48	16.64 ^c^	24.84 ^a^	19.04 ^b^	0.112	<0.0001
72	23.49 ^b^	29.57 ^a^	26.53 ^ab^	1.074	0.020

^1^ Sole substrates of *Chenopodium album* according to harvesting time: June, July, and August; ^2^ standard error of the means; ^3^ dry matter; ^4^ dry matter digestibility; superscripts (a, b, c) indicate statistically significant differences among treatments within the same incubation time.

**Table 6:** Effect of *Chenopodium album* L. sole substrates on in vitro volatile fatty acids (VFAs) production.

Incubation Time (h)	Treatment ^1^			SEM ^2^	*p*-Value
	June	July	August		
Total VFA (mM)					
0	33.01 ^a^	33.16 ^a^	32.20 ^b^	0.089	0.0006
3	41.89 ^a^	41.58 ^ab^	41.19 ^b^	0.129	0.0242
6	39.14 ^a^	39.10 ^a^	37.65 ^b^	0.394	0.0414
9	38.77 ^c^	43.29 ^b^	44.28 ^a^	0.261	<0.0001
12	52.46 ^b^	60.66 ^a^	53.10 ^b^	0.215	<0.0001
24	75.06 ^a^	73.57 ^a^	67.55 ^b^	0.449	<0.0001
48	99.28 ^a^	99.67 ^a^	86.93 ^b^	0.262	<0.0001
72	102.65 ^b^	103.87 ^a^	94.82 ^c^	0.239	<0.0001
Acetate (% of mM)					
0	61.21 ^a^	60.93 ^a^	60.32 ^b^	0.088	0.0010
3	62.92 ^a^	61.45 ^b^	61.80 ^b^	0.146	0.0009
6	61.99	61.00	62.17	0.391	0.1527
9	63.74 ^a^	62.66 ^b^	62.24 ^c^	0.061	<0.0001
12	65.15	65.01	64.86	0.116	0.2669
24	67.35 ^a^	65.70 ^b^	66.18 ^b^	0.245	0.0080
48	66.58 ^a^	66.50 ^a^	65.18 ^b^	0.097	<0.0001
72	66.75 ^a^	65.87 ^b^	65.89 ^b^	0.073	0.0002
Propionate (% of mM)					
0	17.69	17.80	18.14	0.049	0.0016
3	19.25	20.24	19.76	0.103	0.0015
6	18.97	20.18	18.47	0.151	0.0005
9	18.67 ^b^	19.37 ^a^	18.12 ^c^	0.129	0.0014
12	19.31 ^b^	19.77 ^a^	19.27 ^b^	0.081	0.0079
24	18.84 ^b^	20.10 ^a^	18.29 ^b^	0.264	0.0073
48	18.81 ^a^	18.52 ^b^	18.94 ^a^	0.078	0.0241
72	18.53 ^a^	18.59 ^a^	17.98 ^b^	0.099	0.0090
Iso-butyrate (% of mM)					
0	3.36 ^b^	3.37 ^b^	3.45 ^a^	0.012	0.0027
3	2.75 ^b^	2.77 ^ab^	2.79 ^a^	0.010	0.1106
6	2.96	2.97	3.05	0.031	0.1557
9	2.92 ^a^	2.71 ^b^	2.42 ^c^	0.020	<0.0001
12	2.26 ^a^	2.00 ^b^	2.24 ^a^	0.020	0.0001
24	1.76 ^b^	1.81 ^b^	1.94 ^a^	0.014	0.0002
48	1.78 ^b^	1.71 ^c^	1.89 ^a^	0.003	<0.0001
72	1.88 ^a^	1.81 ^b^	1.89 ^a^	0.014	0.0142
Butyrate (% of mM)					
0	11.32 ^b^	11.50 ^a^	11.55 ^a^	0.025	0.0013
3	9.85 ^b^	10.21 ^a^	10.29 ^a^	0.030	<0.0001
6	10.36	10.07	10.42	0.172	0.3739
9	9.16 ^b^	9.94 ^a^	9.19 ^b^	0.104	0.0029
12	8.79 ^b^	9.02 ^a^	9.10 ^a^	0.057	0.0201
24	8.04 ^c^	8.30 ^b^	9.02 ^a^	0.043	<0.0001
48	8.32 ^c^	8.93 ^b^	9.51 ^a^	0.101	0.0005
72	8.14 ^b^	9.22 ^a^	9.31 ^a^	0.045	<0.0001
Iso-valerate (% of mM)					
0	3.57 ^b^	3.58 ^b^	3.65 ^a^	0.008	0.0011
3	2.97	3.00	3.01	0.014	0.1394
6	3.19	3.15	3.24	0.043	0.4181
9	3.01 ^a^	2.89 ^b^	2.54 ^c^	0.024	<0.0001
12	2.40 ^a^	2.21 ^b^	2.40 ^a^	0.009	<0.0001
24	2.13 ^b^	2.11 ^b^	2.49 ^a^	0.028	0.0001
48	2.59 ^b^	2.49 ^c^	2.66 ^a^	0.016	0.0008
72	2.88 ^a^	2.80 ^b^	2.92 ^a^	0.016	0.0023
Valerate (% of mM)					
0	2.86 ^b^	2.83 ^c^	2.89 ^a^	0.005	0.0003
3	2.3	2.34	2.35	0.015	0.1207
6	2.54	2.54	2.65	0.035	0.0989
9	2.48 ^a^	2.42 ^b^	2.19 ^c^	0.015	<0.0001
12	2.08 ^b^	1.95 ^c^	2.13 ^a^	0.006	<0.0001
24	1.92 ^b^	1.96 ^b^	2.10 ^a^	0.022	0.0027
48	1.83 ^b^	1.83 ^b^	1.97 ^a^	0.006	<0.0001
72	1.87 ^b^	1.90 ^b^	1.98 ^a^	0.011	0.0011
A/P ^3^ ratio					
0	3.46 ^a^	3.42 ^a^	3.33 ^b^	0.014	0.0014
3	3.27 ^a^	3.04 ^c^	3.13 ^b^	0.023	0.0011
6	3.27 ^a^	3.02 ^b^	3.37 ^a^	0.047	0.0051
9	3.41 ^b^	3.24 ^b^	3.24 ^b^	0.019	0.0009
12	3.38 ^a^	3.29 ^b^	3.37 ^a^	0.019	0.0345
24	3.58 ^a^	3.27 ^b^	3.62 ^a^	0.063	0.015
48	3.54 ^a^	3.59 ^a^	3.44 ^b^	0.018	0.0034
72	3.60 ^ab^	3.54 ^b^	3.66 ^a^	0.022	0.0213

^1^ Sole substrates of *Chenopodium album* according to harvesting time: June, July, and August; ^2^ standard error of the means; ^3^ acetate to propionate ratio; superscripts (a, b, c) indicate statistically significant differences among treatments within the same incubation time.

**Table 7:** Effect of *Chenopodium album* L. substitution levels on pH, in vitro dry matter digestibility (IVDMD), and ammonia–nitrogen (NH_3_-N) concentration.

**Treatments ^1^**	**Incubation time (h)**							
	3		6	9	12	24	48	72
	pH							
Control	6.76 ^b^		6.75 ^c^	6.67 ^d^	6.74 ^a^	6.55 ^a^	6.54 ^a^	6.45
T1	6.78 ^b^		7.04 ^a^	6.69 ^c^	6.63 ^b^	6.53 ^a^	6.48 ^d^	6.48
T2	6.83 ^a^		6.85 ^b^	6.74 ^b^	6.58 ^c^	6.54 ^a^	6.50 ^c^	6.47
T3	6.82 ^a^		6.86 ^b^	6.83 ^a^	6.59 ^c^	6.53 ^a^	6.50 ^c^	6.46
T4	6.83 ^a^		6.81 ^bc^	6.72 ^bc^	6.57 ^c^	6.48 ^b^	6.53 ^b^	6.46
SEM ^2^	0.01		0.024	0.014	0.009	0.013	0.009	0.014
*p* value	0.0006		<0.0001	0.0002	<0.0001	0.0184	<0.0001	0.4068
L ^3^	<0.0001		0.5247	0.0004	<0.0001	0.0098	0.0004	0.9704
Q ^3^	0.0474		0.0004	0.0038	<0.0001	0.0453	0.7337	0.2620
C ^3^	0.7213		0.0002	0.0007	0.0147	0.1427	<0.0001	0.1792
	IVDMD (%)							
Control	24.27 ^bc^		33.42 ^b^	47.89 ^a^	51.38 ^b^	64.26 ^b^	76.11 ^b^	80.22 ^a^
T1	28.94 ^a^		37.13 ^a^	43.39 ^bc^	54.28 ^a^	65.46 ^ab^	76.70 ^b^	80.90 ^a^
T2	23.57 ^c^		34.57 ^ab^	44.74 ^b^	55.67 ^a^	67.16 ^a^	78.12 ^a^	80.20 ^a^
T3	25.85 ^b^		35.46 ^ab^	48.50 ^a^	54.61 ^a^	67.29 ^a^	76.54 ^b^	80.89 ^a^
T4	14.71 ^d^		27.90 ^c^	42.03 ^c^	44.43 ^c^	59.62 ^c^	71.06 ^c^	73.66 ^b^
SEM ^2^	0.613		0.938	0.605	0.756	0.740	0.382	0.480
*p* value	<0.0001		0.0004	<0.0001	<0.0001	0.0001	<0.0001	<0.0001
L ^3^	<0.0001		0.0016	0.0062	0.0002	0.0099	<0.0001	<0.0001
Q ^3^	<0.0001		0.0003	0.5137	<0.0001	<0.0001	<0.0001	<0.0001
C ^3^	0.1105		0.4812	<0.0001	0.0099	0.0053	0.0028	0.0015
	NH_3_-N (mg/100 mL)							
	0	3	6	9	12	24	48	72
Control	1.57 ^c^	1.77 ^c^	1.18 ^c^	1.43 ^a^	1.77 ^a^	1.75 ^d^	7.76 ^a^	14.87 ^a^
T1	1.51 ^c^	2.97 ^a^	1.60 ^b^	1.26 ^b^	1.57 ^c^	1.88 ^c^	6.09 ^b^	13.91 ^a^
T2	1.75 ^b^	2.14 ^b^	1.55 ^b^	0.83 ^d^	1.69 ^ab^	1.91 ^c^	7.97 ^a^	9.50 ^c^
T3	1.95 ^a^	2.88 ^a^	1.72 ^ab^	1.18 ^b^	1.66 ^b^	2.45 ^a^	7.55 ^a^	11.29 ^b^
T4	1.52 ^c^	2.37 ^b^	1.86 ^a^	1.01 ^c^	1.43 ^d^	2.26 ^b^	7.71 ^a^	14.95 ^a^
SEM ^2^	0.048	0.086	0.073	0.04	0.024	0.038	0.15	0.415
*p* value	0.0003	<0.0001	0.0008	<0.0001	<0.0001	<0.0001	<0.0001	<0.0001
L ^3^	0.0403	0.0022	<0.0001	0.0002	<0.0001	<0.0001	0.0166	0.09
Q ^3^	0.0013	0.0002	0.2464	0.115	0.0387	0.4472	0.0373	<0.0001
C ^3^	<0.0001	0.0159	0.0934	0.0843	<0.0001	0.0004	<0.0001	0.0023

^1^ Substitution levels of *Chenopodium album* harvested in August by percentage: Control (0), T1 (5), T2 (10), T3 (15), and T4 (20); ^2^ standard error of the means; ^3^ L: linear effect, Q: quadratic effect, C: cubic effect; superscripts (a, b, c, d) indicate statistically significant differences among treatments within the same incubation time.

**Table 8:** Effect of *Chenopodium album* L. substitution levels on in vitro total gas (TGP) and methane (CH_4_) production.

**Treatments ^1^**	**Incubation time (h)**						
	3	6	9	12	24	48	72
	TGP (mL/g DM)						
Control	19.14 ^b^	37.86 ^b^	46.57 ^c^	74.57 ^a^	113.33 ^a^	138.27 ^ab^	148.95 ^a^
T1	18.95 ^b^	40.06 ^a^	50.88 ^a^	75.34 ^a^	113.53 ^a^	140.32 ^a^	149.58 ^a^
T2	19.27 ^b^	37.50 ^bc^	48.92 ^ab^	76.01 ^a^	108.38 ^b^	137.17 ^b^	147.86 ^ab^
T3	19.52 ^b^	35.44 ^d^	47.04 ^bc^	71.56 ^b^	107.88 ^b^	136.30 ^b^	146.29 ^b^
T4	20.22 ^a^	35.88 ^cd^	50.29 ^a^	68.88 ^b^	108.82 ^b^	136.10 ^b^	148.05 ^ab^
SEM ^2^	0.204	0.565	0.674	0.954	0.646	0.761	0.646
*p* value	0.0109	0.0013	0.0036	0.0017	0.0001	0.0162	0.041
L ^3^	0.0018	0.0007	0.1232	0.0005	<0.0001	0.006	0.0316
Q ^3^	0.048	0.1808	0.4379	0.0071	0.0297	0.4571	0.3417
C ^3^	0.9138	0.0022	0.0003	0.5515	0.0077	0.0346	0.0193
	CH_4_ (mL/g DM ^4^)						
Control	0.60 ^b^	2.33 ^b^	3.58 ^d^	7.59 ^bc^	24.84 ^a^	42.55 ^a^	51.12 ^ab^
T1	0.57 ^b^	2.47 ^a^	4.17 ^a^	8.01 ^a^	22.79 ^b^	41.21 ^bc^	52.58 ^a^
T2	0.64 ^a^	2.42 ^ab^	3.71 ^c^	7.46 ^bc^	22.19 ^b^	41.95 ^ab^	51.26 ^ab^
T3	0.58 ^b^	2.01 ^c^	3.63 ^cd^	7.25 ^c^	21.61 ^b^	40.43 ^c^	47.09 ^c^
T4	0.63 ^a^	2.47 ^a^	4.03 ^b^	7.64 ^b^	22.44 ^b^	40.37 ^c^	50.63 ^b^
SEM ^2^	0.0086	0.037	0.034	0.116	0.425	0.410	0.569
*p* value	0.0005	<0.0001	<0.0001	0.0113	0.003	0.0142	0.0005
L ^3^	0.0515	0.1117	0.0075	0.1044	0.0012	0.0027	0.0048
Q ^3^	0.4342	0.0753	0.817	0.5347	0.0047	0.8579	0.5487
C ^3^	0.3912	<0.0001	<0.0001	0.0018	0.9718	0.6440	0.0002
	CH_4_ (mL/g DMD ^5^)						
Control	2.48 ^b^	6.99 ^b^	7.53 ^d^	14.33 ^b^	36.09 ^b^	55.84 ^a^	63.86 ^a^
T1	1.97 ^c^	7.24 ^b^	8.54 ^b^	14.93 ^b^	35.11 ^b^	54.11 ^ab^	65.09 ^a^
T2	2.44 ^b^	7.15 ^b^	8.09 ^c^	13.42 ^c^	33.05 ^c^	54.69 ^ab^	60.46 ^bc^
T3	2.13 ^bc^	5.52 ^c^	7.42 ^d^	14.69 ^b^	32.10 ^c^	52.70 ^b^	59.23 ^c^
T4	3.34 ^a^	8.84 ^a^	9.13 ^a^	16.28 ^a^	38.63 ^a^	55.63 ^a^	62.67 ^ab^
SEM ^2^	0.141	0.168	0.09	0.277	0.495	0.72	0.945
*p* value	0.0004	<0.0001	<0.0001	0.0004	<0.0001	0.0659	0.0077
L ^3^	0.0018	0.0039	<0.0001	0.0019	0.2176	0.4428	0.0203
Q ^3^	0.0005	<0.0001	0.0058	0.001	<0.0001	0.0309	0.0515
C ^3^	0.2586	<0.0001	<0.0001	0.0196	0.0003	0.2756	0.0055

^1^ Substitution levels of *Chenopodium album* harvested in August by percentage: Control (0), T1 (5), T2 (10), T3 (15), and T4 (20); ^2^ standard error of the means; ^3^ L: linear effect, Q: quadratic effect, C: cubic effect; ^4^ dry matter; ^5^ dry matter digestibility; superscripts (a, b, c, d) indicate statistically significant differences among treatments within the same incubation time.

**Table 9:** Effect of *Chenopodium album* L. substitution levels on in vitro volatile fatty acids (VFAs) production.

**Treatments ^1^**	**Incubation time (h)**							
	0	3	6	9	12	24	48	72
	Total VFA (mM)							
Control	20.52 ^a^	34.08 ^a^	40.88 ^c^	46.67 ^c^	63.47 ^a^	88.52 ^b^	81.20 ^c^	102.91 ^b^
T1	20.86 ^a^	32.37 ^b^	46.44 ^ab^	51.39 ^ab^	56.75 ^b^	85.60 ^b^	78.77 ^c^	100.86 ^b^
T2	20.29 ^ab^	32.14 ^b^	42.35 ^bc^	52.38 ^a^	59.33 ^ab^	98.28 ^a^	85.72 ^b^	104.04 ^ab^
T3	19.46 ^b^	31.85 ^b^	42.34 ^bc^	47.79 ^bc^	62.20 ^a^	86.66 ^b^	78.86 ^c^	100.17 ^b^
T4	20.67 ^a^	33.92 ^a^	50.35 ^a^	52.68 ^a^	62.32 ^a^	82.66 ^b^	91.35 ^a^	109.05 ^a^
SEM ^2^	0.27	0.455	1.343	1.346	1.336	3.075	1.003	1.776
*p* value	0.0336	0.0147	0.0031	0.0293	0.0301	0.0413	<0.0001	0.0364
L ^3^	0.2198	0.5802	0.0057	0.0759	0.4738	0.2996	<0.0001	0.066
Q ^3^	0.1797	0.0013	0.1039	0.3226	0.0191	0.0443	0.0016	0.0501
C ^3^	0.0063	0.5583	0.0019	0.011	0.0173	0.4305	0.0104	0.2108
	Acetate (% of mM)							
Control	59.50 ^a^	58.89 ^d^	57.13 ^ab^	56.50 ^c^	58.04 ^c^	58.53 ^a^	55.61 ^bc^	54.41 ^a^
T1	59.39 ^a^	58.93 ^d^	56.65 ^ab^	57.68 ^bc^	59.05 ^a^	58.33 ^a^	56.26 ^a^	54.18 ^ab^
T2	59.38 ^a^	59.23 ^c^	57.72 ^a^	59.27 ^ab^	59.12 ^a^	58.25 ^ab^	55.98 ^ab^	53.90 ^b^
T3	59.31 ^a^	59.66 ^b^	57.62 ^a^	59.41 ^a^	58.71 ^ab^	57.40 ^c^	55.82 ^abc^	52.58 ^c^
T4	58.69 ^b^	60.04 ^a^	56.37 ^b^	58.35 ^ab^	58.46 ^bc^	57.52 ^bc^	55.45 ^c^	52.30 ^c^
SEM ^2^	0.126	0.096	0.346	0.54	0.153	0.24	0.165	0.154
*p* value	0.0076	<0.0001	0.0786	0.0182	0.0029	0.024	0.0436	<0.0001
L ^3^	0.0017	<0.0001	0.6291	0.0098	0.3232	0.0031	0.1707	<0.0001
Q ^3^	0.0449	0.0482	0.0623	0.015	0.0003	0.8981	0.0108	0.0754
C ^3^	0.1329	0.331	0.0328	0.3706	0.0443	0.2778	0.2097	0.0459
	Propionate (% of mM)							
Control	15.93 ^b^	20.31 ^a^	23.60 ^b^	24.73 ^ab^	25.39 ^a^	25.76 ^c^	27.75 ^b^	31.65 ^b^
T1	16.30 ^a^	19.74 ^b^	24.36 ^ab^	24.83 ^ab^	24.25 ^c^	25.62 ^c^	26.85 ^c^	32.02 ^b^
T2	16.00 ^b^	19.54 ^b^	23.57 ^b^	24.40 ^bc^	24.51 ^bc^	26.36 ^b^	27.90 ^b^	32.34 ^b^
T3	15.86 ^b^	19.40 ^b^	23.78 ^b^	24.25 ^c^	25.08 ^ab^	27.21 ^a^	27.73 ^b^	32.52 ^ab^
T4	16.19 ^a^	19.62 ^b^	25.14 ^a^	25.13 ^a^	25.06 ^ab^	27.51 ^a^	29.54 ^a^	33.46 ^a^
SEM ^2^	0.06	0.125	0.291	0.138	0.233	0.168	0.227	0.326
*p* value	0.002	0.004	0.0149	0.008	0.035	<0.0001	0.0001	0.027
L ^3^	0.6369	0.0015	0.0212	0.6341	0.8193	<0.0001	<0.0001	0.0025
Q ^3^	0.7014	0.0058	0.071	0.0052	0.0146	0.1522	0.0006	0.4342
C ^3^	0.0001	0.9847	0.015	0.0051	0.0224	0.0215	0.9717	0.4481
	Iso-butyrate (% of mM)							
Control	5.13 ^b^	3.33 ^bc^	2.86 ^a^	2.57 ^a^	1.98 ^b^	1.55 ^c^	1.87 ^a^	1.62 ^ab^
T1	5.15 ^b^	3.45 ^ab^	2.59 ^b^	2.32 ^b^	2.15 ^a^	1.67 ^a^	1.92 ^a^	1.65 ^a^
T2	5.11 ^b^	3.46 ^a^	2.76 ^ab^	2.27 ^b^	2.03 ^b^	1.46 ^d^	1.80 ^b^	1.62 ^ab^
T3	5.29 ^a^	3.47 ^a^	2.75 ^ab^	2.35 ^b^	2.01 ^b^	1.61 ^b^	1.92 ^a^	1.68 ^a^
T4	5.17 ^b^	3.29 ^c^	2.38 ^c^	2.27 ^b^	2.04 ^b^	1.61 ^b^	1.67 ^c^	1.57 ^b^
SEM ^2^	0.027	0.041	0.058	0.058	0.027	0.013	0.023	0.019
*p* value	0.0057	0.0283	0.0012	0.0225	0.0134	<0.0001	<0.0001	0.0278
L ^3^	0.04	0.7124	0.0013	0.0104	0.8631	0.2798	0.0003	0.2942
Q ^3^	0.5856	0.0026	0.1135	0.0659	0.1248	0.0238	0.0021	0.0173
C ^3^	0.014	0.4884	0.0012	0.0827	0.0017	0.0022	0.0143	0.0688
	Butyrate (% of mM)							
Control	9.97 ^bc^	11.07 ^a^	10.73 ^ab^	10.97 ^a^	10.22 ^a^	10.26 ^a^	9.81 ^a^	8.04 ^a^
T1	10.34 ^ab^	11.07 ^a^	10.58 ^ab^	10.22 ^ab^	9.96 ^c^	10.21 ^a^	9.93 ^a^	7.89 ^b^
T2	9.59 ^c^	11.03 ^ab^	10.47 ^b^	9.56 ^b^	9.78 ^d^	10.01 ^b^	9.73 ^a^	7.49 ^d^
T3	10.14 ^b^	10.87 ^bc^	10.40 ^b^	9.28 ^b^	9.78 ^d^	9.90 ^c^	9.39 ^b^	7.58 ^c^
T4	10.65 ^a^	10.84 ^c^	10.98 ^a^	9.69 ^b^	10.09 ^b^	9.69 ^d^	8.94 ^c^	7.59 ^c^
SEM ^2^	0.147	0.058	0.146	0.305	0.027	0.03	0.066	0.02
*p* value	0.0051	0.0421	0.1074	0.0209	<0.0001	<0.0001	<0.0001	<0.0001
L ^3^	0.0314	0.0046	0.4949	0.0047	0.0004	<0.0001	<0.0001	<0.0001
Q ^3^	0.0167	0.3786	0.0198	0.0405	<0.0001	0.0495	0.0005	<0.0001
C ^3^	0.0443	0.3382	0.2069	0.5521	0.0337	0.5571	0.3162	0.0289
	Isovalerate (% of mM)							
Control	5.06 ^c^	3.55 ^c^	3.15 ^a^	2.92 ^a^	2.36 ^bc^	1.98 ^b^	2.63 ^c^	2.36 ^c^
T1	5.13 ^bc^	3.72 ^ab^	2.95 ^c^	2.61 ^b^	2.46 ^a^	2.07 ^a^	2.71 ^a^	2.36 ^c^
T2	5.05 ^c^	3.76 ^a^	3.06 ^b^	2.54 ^b^	2.35 ^c^	1.90 ^c^	2.48 ^d^	2.39 ^b^
T3	5.22 ^a^	3.68 ^b^	3.08 ^ab^	2.57 ^b^	2.29 ^d^	2.01 ^b^	2.67 ^b^	2.42 ^a^
T4	5.17 ^ab^	3.55 ^c^	2.84 ^d^	2.51 ^b^	2.39 ^d^	2.01 ^b^	2.32 ^e^	2.32 ^d^
SEM ^2^	0.03	0.016	0.026	0.085	0.011	0.013	0.012	0.007
*p* value	0.0092	<0.0001	<0.0001	0.0397	<0.0001	<0.0001	<0.0001	<0.0001
L ^3^	0.007	0.6046	0.0001	0.01	0.0042	0.8061	<0.0001	0.3144
Q ^3^	0.9893	<0.0001	0.1423	0.0862	0.227	0.1156	<0.0001	<0.0001
C ^3^	0.4121	0.1715	<0.0001	0.2497	<0.0001	0.0119	<0.0001	<0.0001
	Valerate (% of mM)							
Control	3.99 ^d^	2.71 ^c^	2.48 ^a^	1.88 ^b^	2.00 ^d^	1.79 ^d^	2.30 ^c^	2.07 ^c^
T1	4.03 ^c^	2.85 ^ab^	2.35 ^b^	2.11 ^a^	2.23 ^a^	2.00 ^a^	2.49 ^a^	2.08 ^c^
T2	4.01 ^cd^	2.89 ^a^	2.43 ^a^	2.07 ^a^	2.07 ^c^	1.87 ^c^	2.35 ^b^	2.10 ^c^
T3	4.16 ^a^	2.84 ^b^	2.44 ^a^	2.11 ^a^	2.00 ^d^	1.92 ^b^	2.49 ^a^	2.57 ^a^
T4	4.07 ^b^	2.71 ^c^	2.06 ^c^	2.05 ^ab^	2.11 ^b^	2.03 ^a^	2.11 ^d^	2.26 ^b^
SEM ^2^	0.009	0.014	0.018	0.056	0.011	0.013	0.006	0.03
*p* value	<0.0001	<0.0001	<0.0001	0.0754	<0.0001	<0.0001	<0.0001	<0.0001
L ^3^	<0.0001	0.9185	<0.0001	0.0858	0.7714	<0.0001	<0.0001	<0.0001
Q ^3^	0.0176	<0.0001	<0.0001	0.0362	0.0038	0.5233	<0.0001	0.1133
C ^3^	<0.0001	0.5334	<0.0001	0.3659	<0.0001	<0.0001	<0.0001	<0.0001
	A/P ^4^ ratio							
Control	3.76 ^a^	2.90 ^e^	2.42 ^ab^	2.29 ^b^	2.31 ^c^	2.27 ^a^	2.00 ^b^	1.72 ^a^
T1	3.64 ^c^	2.99 ^d^	2.36 ^b^	2.31 ^b^	2.44 ^a^	2.26 ^a^	2.07 ^a^	1.69 ^a^
T2	3.71 ^ab^	3.03 ^c^	2.45 ^a^	2.39 ^ab^	2.42 ^ab^	2.21 ^a^	2.01 ^ab^	1.66 ^a^
T3	3.71 ^ab^	3.12 ^a^	2.42 ^ab^	2.42 ^ab^	2.31 ^c^	2.11 ^b^	2.01 ^ab^	1.65 ^a^
T4	3.69 ^b^	3.06 ^b^	2.47 ^a^	2.79 ^a^	2.36 ^bc^	2.08 ^b^	1.87 ^c^	1.55 ^b^
SEM ^2^	0.017	0.01	0.025	0.129	0.021	0.022	0.02	0.028
*p* value	0.0064	<0.0001	0.0752	0.1094	0.0036	0.0002	0.0006	0.0169
L ^3^	0.201	<0.0001	0.0399	0.0196	0.7801	<0.0001	0.0004	0.0013
Q ^3^	0.0806	<0.0001	0.4375	0.2159	0.0158	0.3203	0.001	0.2602
C ^3^	0.003	0.0073	0.3634	0.5213	0.001	0.1512	0.7817	0.4031

^1^ Substitution levels of *Chenopodium album* harvested in August by percentage: Control (0), T1 (5), T2 (10), T3 (15), and T4 (20); ^2^ standard error of the means; ^3^ L: linear effect, Q: quadratic effect, C: cubic effect; ^4^ acetate to propionate ratio; superscripts (a, b, c, d, e) indicate statistically significant differences among treatments within the same incubation time.

## Data Availability

The raw data supporting the conclusions of this article will be made available by the authors on request.

## Supplementary Materials

The following supporting information can be downloaded at: https://www.mdpi.com/article/10.3390/ani15101372/s1, Figure S1: Effect of harvest time of sole-substrate *Chenopodium album* L. (CAL) on in vitro rumen fermentation parameters over time. (A) pH; (B) In vitro dry matter digestibility (IVDMD); (C) Total gas production; (D) Methane (CH₄) production; (E) Total volatile fatty acids (TVFA); (F) Ammonia nitrogen (NH₃-N) concentration. Values are presented as means ± SEM (n = 3); Figure S2: Effect of *Chenopodium album* L. substitution levels on in vitro rumen fermentation parameters over time. (A) pH; (B) In vitro dry matter digestibility (IVDMD); (C) Total gas production; (D) Methane (CH₄) production; (E) Total volatile fatty acids (TVFA); (F) Ammonia nitrogen (NH₃-N) concentration. Values are presented as means ± SEM (n = 3).

## Abbreviations

The following abbreviations are used in this manuscript:

CAL	*Chenopodium Album L.*
CP	Crude protein
NDF	Neutral detergent fiber
ADF	Acid detergent fiber
IVDMD	In vitro dry matter digestibility
A/P	Acetate to propionate
TVFA	Total volatile fatty acids
GHG	Greenhouse gas
CO_2_	Carbon dioxide
CH_4_	Methane
GE	Gross energy
OM	Organic matter
DM	Dry matter
O_2_	Oxygen
TGP	Total gas production
NH_3_-N	Ammonia–nitrogen
EE	Ether extract
CF	Crude fiber
CA	Crude ash
USA	United States of America
SAS	Statistical analysis system
GLM	General linear model
SEM	Standard error of the mean
CNU	Chungnam National University
IPET	Korea Institute of Planning and Evaluation for Technology in Food, Agriculture, and Forestry
MAFRA	Ministry of Agriculture, Food and Rural Affairs, Korea
KOICA	Korea International Cooperation Agency
HKNU	Hankyong National University
