# Supplementary material for: Effects of Chenopodium album L. Substitution Levels and Harvest Time on In Vitro Rumen Fermentation and Methane Production in Early-Fattening Hanwoo Steers
Source: Animals (Basel). 2025 May 9;15(10):1372. doi: 10.3390/ani15101372 (PMC12108182; doi:10.3390/ani15101372)
Supplement: Supplementary file 1 [file animals-15-01372-s001.zip › animals-3596422-supplementary.pdf]

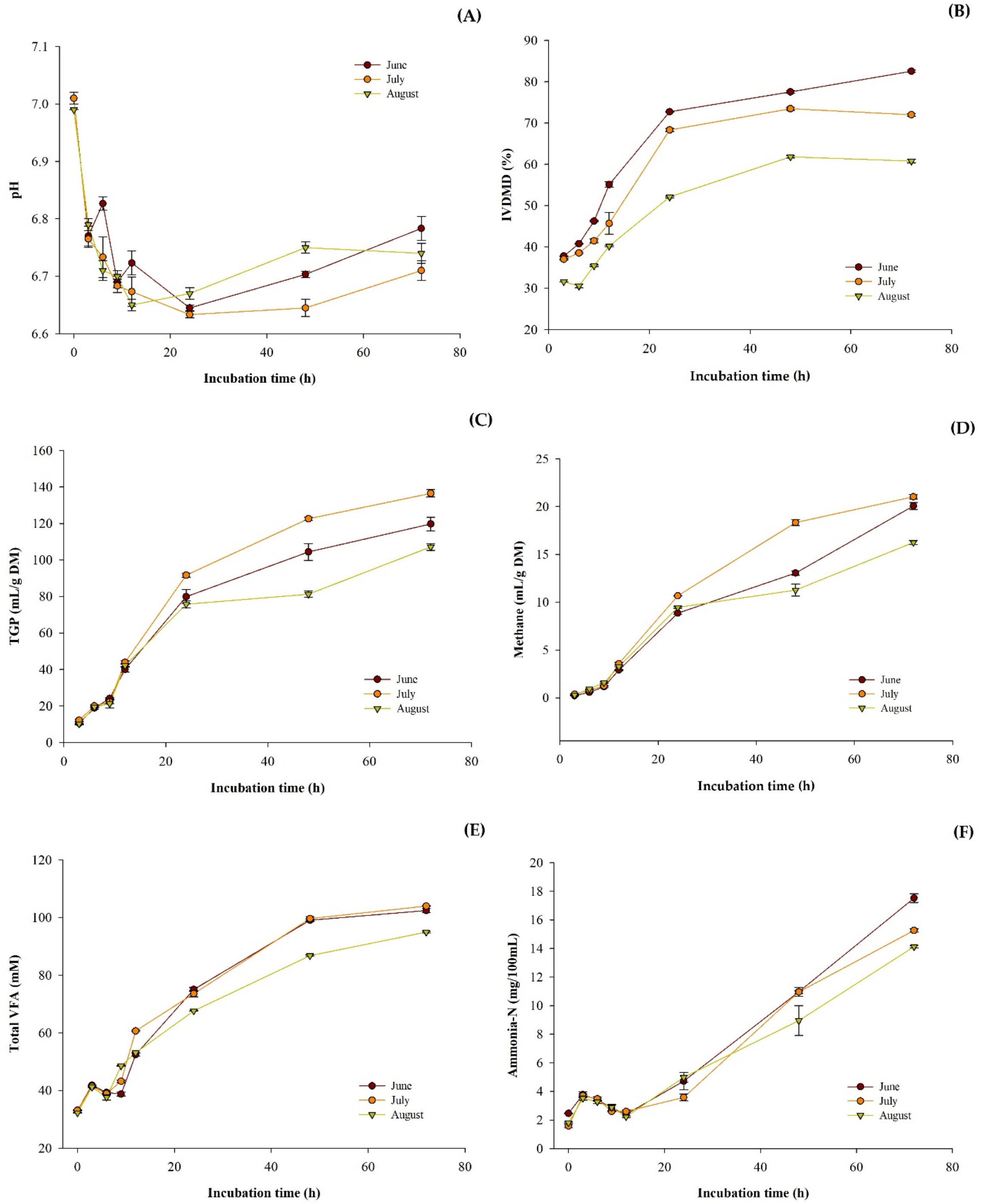

Figure S1: Effect of harvest time of sole-substrate *Chenopodium album* L. (CAL) on in vitro rumen fermentation parameters over time. (A) pH; (B) In vitro dry matter digestibility (IVDMD); (C) Total gas production; (D) Methane (CH_4) production; (E) Total volatile fatty acids (TVFA); (F) Ammonia nitrogen (NH_3-N) concentration. Values are presented as means ± SEM (n = 3).

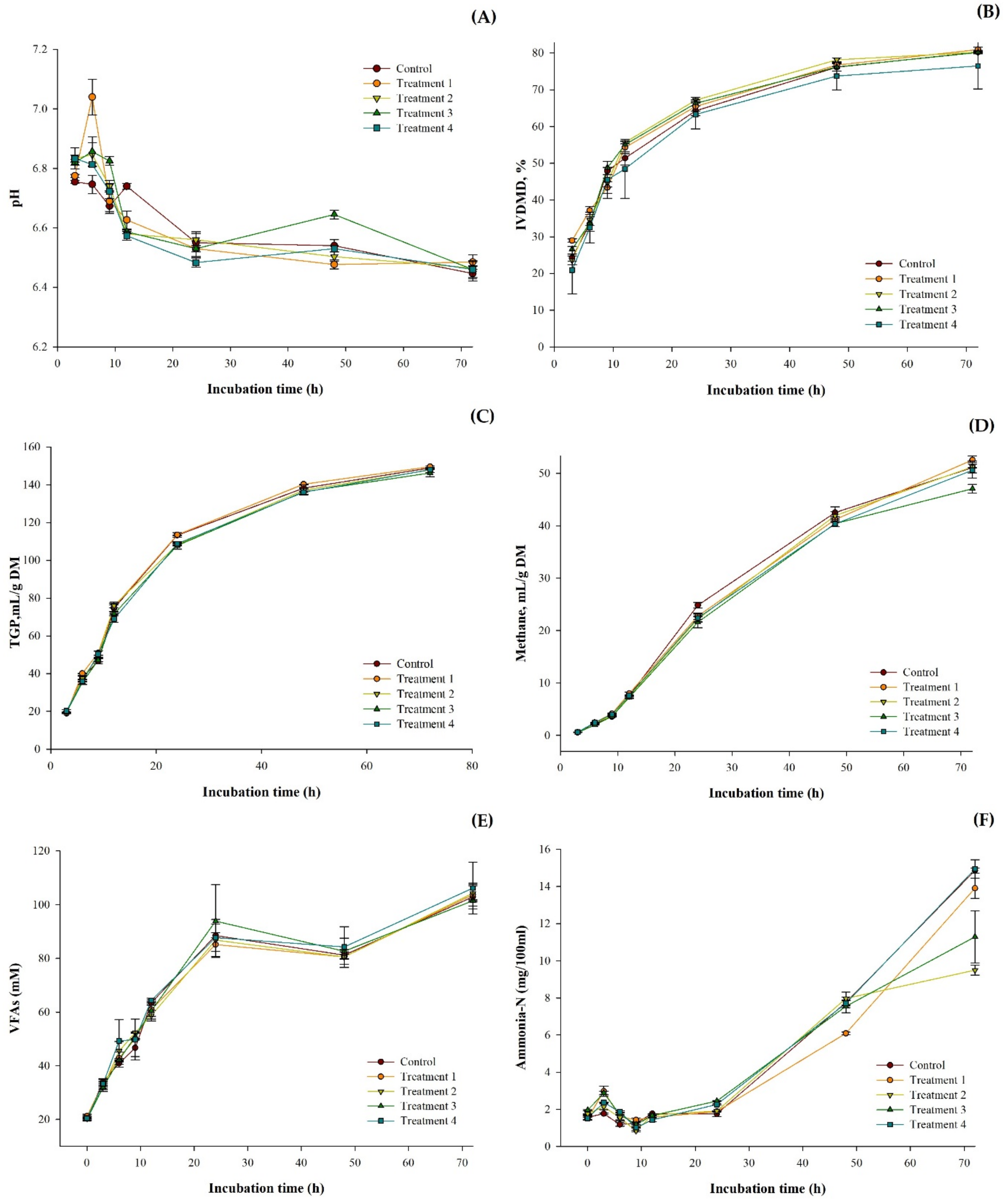

Figure S2: Effect of *Chenopodium album* L. substitution levels on in vitro rumen fermentation parameters over time. (A) pH; (B) In vitro dry matter digestibility (IVDMD); (C) Total gas production; (D) Methane (CH_4) production; (E) Total volatile fatty acids (TVFA); (F) Ammonia nitrogen (NH_3-N) concentration. Values are presented as means ± SEM (n = 3).
